# Gamma-Delta Large Granular Lymphocytic Leukemia: A Diagnostic Dilemma

**DOI:** 10.7759/cureus.24124

**Published:** 2022-04-13

**Authors:** Melissa Oye, Ahmad Alkhasawneh, JR Quan

**Affiliations:** 1 Department of Medicine, University of Florida College of Medicine – Jacksonville, Jacksonville, USA; 2 Department of Pathology, University of Florida College of Medicine – Jacksonville, Jacksonville, USA; 3 Department of Oncology, University of Florida College of Medicine – Jacksonville, Jacksonville, USA

**Keywords:** lymphopenia, hemolytic anemia, t cell neoplasm, lymphocytic leukemia, ttp

## Abstract

We report an initial diagnostic dilemma case of a 60-year-old male who presented with worsening hemolytic anemia, thrombocytopenia, and acute kidney injury requiring hemodialysis. His presentation was initially suspected to be secondary to thrombotic thrombocytopenic purpura (TTP) and he was treated with intravenous immunoglobulin (IVIG) and plasmapheresis. Despite treatment, he failed to improve during his admission leading to further workup revealing gamma-delta T-cell large granular lymphocytic (γδ T-LGL) leukemia. In this paper, we will discuss the features, workup, and treatment of this rare malignancy.

## Introduction

T-cell large granular lymphocyte (T-LGL) leukemia is a chronic mature T-cell malignancy caused by cytotoxic T-cell proliferation infiltrating various organs [1]. The median age of diagnosis is 60-years-old and there is no established gender predilection [1]. Overall, T-LGL has a 10-year survival rate of close to 80% and approximately 25%-50% of patients will not require therapy [2]. Due to the non-specific nature of symptom presentation, diagnosis can be difficult to establish. We report the case of a 60-year-old male who presented with fatigue and weakness and was later diagnosed with γδ T-LGL leukemia. 

## Case presentation

This presentation begins with a 60-year-old African American male with a past medical history of stage 4 chronic kidney disease (CKD-IV), hypertension, and hepatitis C Virus who presented to the emergency room with progressive fatigue and generalized weakness. On presentation, he was found to be tachycardic, tachypneic, and mildly hypotensive. Physical exam revealed pallor and was negative for palpable lymphadenopathy. Complete blood count revealed leukopenia (2,330 leukocytes/µL) with 70% lymphocytes and absolute neutrophil count (ANC) of 1,200, anemia with Hemoglobin of 9.5 g/dL, and thrombocytopenia (70,000 µL). A comprehensive metabolic panel was significant for acute kidney injury on CKD-IV (Cr 6.14 mg/dL). There were initial concerns for sepsis and he was empirically started on broad-spectrum antibiotics. After an extensive infectious workup was negative, antibiotics were discontinued. Autoimmune workup including antinuclear antibody, rheumatoid factor, and C3/C4 were negative. Direct antiglobulin testing (DAT) was positive.

**Figure 1:**
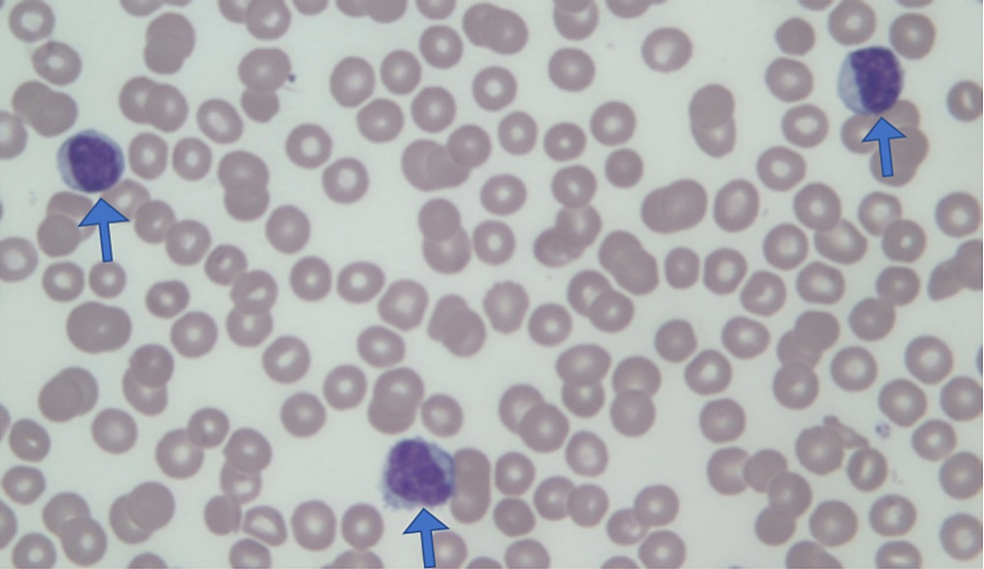
Peripheral blood smear shows lymphocytosis with three large granular lymphocytes (arrows). Giemsa stain (630x).

**Figure 2:**
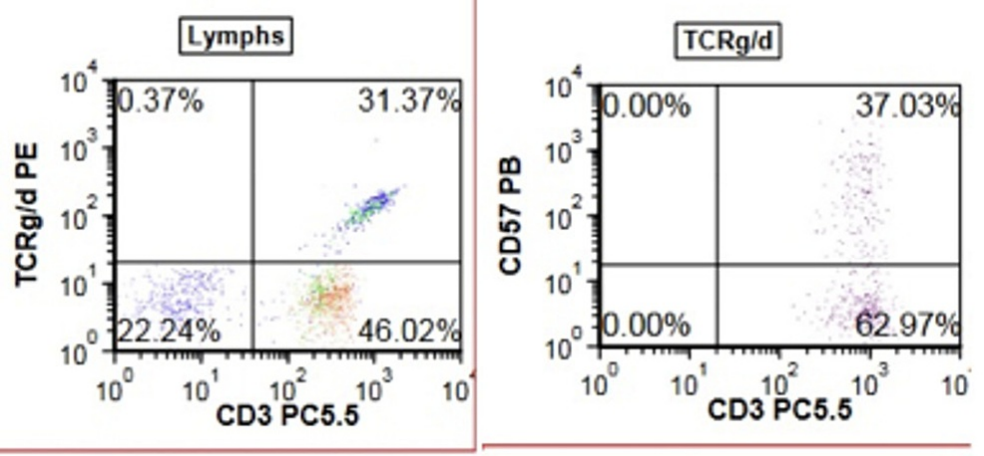
Flow cytometry shows increased circulating γ-δ T- lymphocytes (≈one third of T-cells) (left) and the γ-δ cells show expression of CD57 (right).

**Figure 3:**
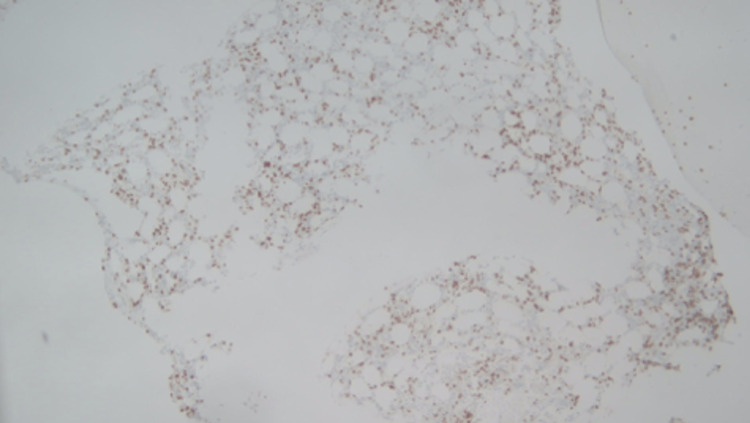
CD3 highlights scattered T-cells with focal aggregates (immunohistochemical stains, 50x).

**Figure 4:**
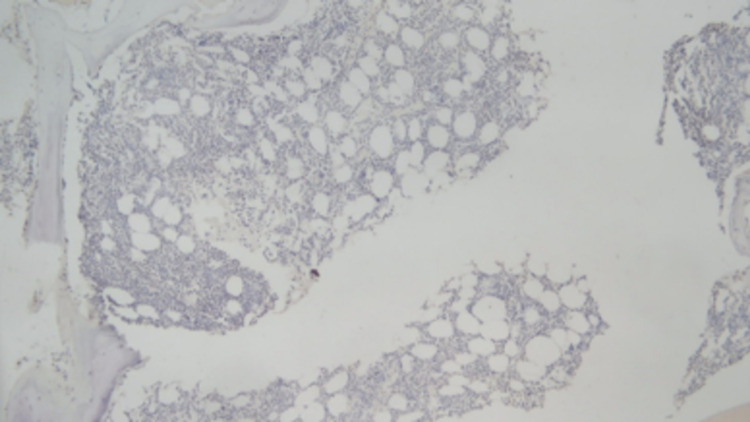
There is no significant expression of TCR Beta F1 cells (immunohistochemical stains, 50x).

Throughout the course of his hospitalization, he became progressively more anemic and thrombocytopenic. He also developed uremia with encephalopathy ultimately requiring hemodialysis. Due to concern for thrombotic thrombocytopenic purpura (TTP), he was started on a trial of intravenous immunoglobulin (IVIG) but had no clinical response. Plasmapheresis was subsequently initiated and then discontinued after ADAMTS13 activity level was inconsistent with TTP. He was started on methylprednisolone and then transitioned to a gradual prednisone taper. A hematopathologist review of the peripheral smear showed lymphocytosis with increased large granular lymphocytes (LGLs) (Figure 1). Subsequently, he underwent a bone marrow biopsy. Flow cytometry of the marrow demonstrated increased circulating gamma-delta T- lymphocytes with dim CD5 expression, partial expression of CD57 in 7% of cells, and CD8 in 18% of cells with no significant expression of CD56 or CD16 (Figure 2). CD3 T-cells showed interstitial distribution with two small aggregates (Figure 3). T-cell receptor (TCR) beta F1 cells were virtually absent among CD3 T-cells (Figure 4). Molecular studies performed at an outside laboratory were positive for gamma-delta T-cell rearrangement indicating marrow involvement by a T-cell lymphoid neoplasm compatible with γδ-T-LGL leukemia. The patient was continued on a prednisone taper and discharged to a short-term rehabilitation facility. On outpatient follow-up three months later, his hemoglobin and platelet levels had normalized.

## Discussion

T-LGL is a chronic lymphoproliferative disorder caused by cytotoxic T-cell clonal proliferation invading multiple organs, including the bone marrow causing cytopenias [1]. A modest lymphocytosis is often seen [1]. LGLs are defined as WBCs measuring 15-18μm with round or kidney-shaped nuclei and abundant cytoplasm containing azurophilic granules [3]. In unaffected individuals, 10%-15% of peripheral blood mononuclear cells are LGLs [3].

T-LGL leukemia is rare and represents less than 5% of all mature lymphocytic leukemias [4]. The course tends to be indolent and approximately one-third of patients are asymptomatic at diagnosis [3]. The median age of diagnosis is 60 years old and it affects both genders equally [1]. The 10-year survival is favorable and up to half of the patients may not require treatment [2]. The pathogenesis of T-LGL is not well defined but is thought to arise from sustained immune stimulation promoting activation of survival signaling pathways that deregulates apoptosis and evades activation-induced cell death [3].

Neoplastic T-LGL cells most commonly infiltrate the bone marrow and spleen. Bone marrow involvement is characterized by lymphoid aggregates in the interstitial and intrasinusoidal areas [1]. Splenic involvement is characterized by splenomegaly and small lymphocytic infiltration with a dense chromatic concentration in the red pulp, follicular hyperplasia, and infiltration of the sinuses [1]. Lymph node involvement is rare. The diagnosis of T-LGL should be a differential diagnosis in patients with unexplained cytopenias and elevated LGLs. Notably, not all LGL proliferations are malignant in etiology. Benign proliferation can be observed in immunosuppressed patients [1]. A definitive diagnosis is established by demonstrating abnormal CD8+ T-cell clonal proliferation. Methods to establish clonality include polymerase chain reaction (PCR) and Southern blotting. PCR is the process most commonly used with a sensitivity of up to 80% [1]. Southern blotting yields results with higher accuracy, however, is labor-intensive and requires a large amount of good quality DNA [1]. 

The vast majority of patients show a classic immunophenotype pattern of CD3+, CD8+, CD16+, CD57+, CD4-, CD56- [1]. The TCR is often of the αβ subtype; however, our patient had negative TCR Beta F1 cells suggesting against αβ TCR subtype. Ultimately, he was found to have a rarer TCR subtype of γδ. We will now review the immunophenotypic differences of αβ T-LGL and γδ T-LGL leukemia. TCR γδ T-cells are typically a small proportion of peripheral lymphocytes, up to 5% [5]. Therefore, unsurprisingly, the γδ variant of T-LGL is fairly rare. In contrast to the well-established immunophenotype pattern of αβ T-LGL, γδ T-LGL leukemia is more variable. Approximately 40% of patients demonstrate double negative CD4 and CD8; other cases show CD4- and CD8+ with dim expression [5]. CD16 and CD56 show variable expression, while CD57+ is common like in the αβ subtype [5]. 

Given that αβ T-LGL and γδ T-LGL have similar clinical presentations, treatment and prognoses, we will speak of them as one entity moving forward unless otherwise specified. T-LGL commonly presents as neutropenia, anemia, and interestingly rheumatoid arthritis (RA) [6]. Recurrent bacterial infections secondary to severe neutropenia may be the initial presentation. Skin infections, abscesses, and respiratory infections are commonly seen [1]. Nearly half of patients develop severe neutropenia as defined by ANC < 500/μL [6]. Up to 35% of patients may develop transfusion-dependent anemia secondary to autoimmune hemolysis or acquired pure red cell aplasia [4]. There is a well-established association between T-LGL and autoimmune disorders [7,8]. In an evaluation of 44 patients with γδ T-LGL, 34% had a concomitant autoimmune disorder with rheumatoid arthritis having the highest prevalence of 16% [7]. RA may be diagnosed prior to T-LGL [3]. 

Like other hematological malignancies, not all patients require immediate treatment. Some indications to initiate treatment include symptomatic cytopenias and the presence of associated auto-immune conditions [8]. While no guidelines exist on standard treatment, the basis of therapy is immunosuppression with agents like methotrexate, cyclophosphamide, and cyclosporine [8]. Other therapies include fludarabine with mitoxantrone and dexamethasone [9]. Splenectomy has limited results, but has been completed for treatment of neoplasm- associated ITP [10]. In patients with refractory disease, purine analogs, alemtuzumab, or splenectomy can be considered [9]. Our patient responded well to mono-steroid therapy and did not require addition of other immunosuppressants on three- and six-month follow-up examination. There have been some case reports of spontaneous remission [11].

## Conclusions

T-LGL leukemia is a chronic lymphoproliferative disorder that typically follows an indolent course with a favorable 10-year survival rate. The non-specific symptoms, hematologic abnormalities, and association with autoimmune disorders can make the initial diagnosis challenging or even mimic other conditions. Given its rarity, T-LGL leukemia is often not a primary differential diagnosis. We hope from this case report, readers take away the valuable information a cell count differential can provide. While CBC abnormalities are quite routine in acutely ill hospitalized patients, sometimes they represent an undiagnosed malignancy.
